# Detection of Canine Vector-Borne Filariasis and Their *Wolbachia* Endosymbionts in French Guiana

**DOI:** 10.3390/microorganisms8050770

**Published:** 2020-05-21

**Authors:** Younes Laidoudi, Jean-Lou Marié, Djamel Tahir, Stéphanie Watier-Grillot, Oleg Mediannikov, Bernard Davoust

**Affiliations:** 1 Aix Marseille Univ., IRD, AP-HM, MEPHI, IHU-Méditerranée Infection, 13385 Marseille, France; younes.laidoudi@yahoo.com (Y.L.); djamel.tahir@yahoo.fr (D.T.); olegusss1@gmail.com (O.M.); 2 IHU Méditerranée Infection, 13385 Marseille, France; 3 Animal Epidemiology Working Group of the Military Health Service, 13014 Marseille, France; jean-lou.marie@wanadoo.fr (J.-L.M.); s.watier.grillot@gmail.com (S.W.-G.); 4 Epidemiology and Public Health Center of the French Armed Forces, 13014 Marseille, France

**Keywords:** canine vector-borne helminth, filariasis, *Wolbachia*, species diversity, zoonosis, French Guiana

## Abstract

In French Guiana, canine heartworm disease is well known, but the diversity of filarial parasites of dogs remains largely unknown. A total of 98 canine blood samples from Cayenne and Kourou were assessed by a blood wet mount preparation, heartworm antigen test and molecular exploration of filarioid and *Wolbachia* DNAs, followed by a multiplex species-specific qPCR’s identification and a subsequent sequencing analysis. Thereafter, a phylogeny based on maximum likelihood was carried out to facilitate specific identification. Five dogs were microfilaremic. Heartworm antigens were detected in 15 (15.3%) dogs. Of these, six (6.1%) were considered as occult infections as neither microfilariae nor *Dirofilaria immitis* DNA were detected. The 11 (11.2%) *D. immitis* isolates corresponded to a low virulent strain. Six of the *D. immitis* isolates were positive for *Wolbachia* endosymbionts of *D. immitis* belonging to the clade C DNA. *Acanthocheilonema reconditum* DNA was detected in 3 (3.1%) samples. Of these latter, one was found co-infected with the *Brugia* sp. genotype and the DNA of the clade D of the *Wolbachia* endosymbiont of *Brugia* species. This latter was also detected in two filarioid DNA-free samples. Finally, two samples were positive for *Cercopithifilaria bainae* genotype, which is distinct from those identified in Europe. The present study highlights the urgent need to implement chemoprophylaxis associated with anti-*Wolbachia* drugs to control these potential zoonoses.

## 1. Introduction

Canine vector-borne diseases (CVBDs) constitute a worldwide group of illnesses affecting dogs. They are caused by a wide range of bacteria, viruses, protozoa and helminths, all transmitted to dogs by parasitic arthropods bites [1]. These diseases constitute an important public health concern in tropical and subtropical regions from the Old and New World, where they are endemic [2]. In French Guiana, dogs seem to be affected by several major CVBDs, such as leishmaniasis [3], anaplasmosis [4], trypanosomiasis [5], ehrlichiosis [6] and arbovirosis [7].

Canine filariasis are a group of canine vector-borne helminth (CVBH) caused by several nematodes belonging to the Onchocercidae family. Canids constitute suitable hosts for many filarial parasites of veterinary and human importance, such as the zoonotic *Dirofilaria immitis* (Leidy 1856), the agent of cardiopulmonary dirofilariasis (also known as heartworm) in dogs and pulmonary dirofilariasis in human [8], *D. repens* which causes in humans, like in dogs the subcutaneous filariasis [9]. These two species, together with brugian parasites that cause lymphatic filariasis (e.g., *Brugia timori*, *B. malayi* and *B. pahangi*) [10,11], constitute the most thread-like filarial worms causing millions of canine and human cases throughout the world [12,13,14]. These parasites have bloodstream microfilariae and are transmitted by mosquito bites. Dogs may also be affected with another less or completely avirulent group of CVBH transmitted by parasitic arthropods other than mosquitoes, such as *Acanthocheilonema reconditum* and *Cercopithifilaria* spp. parasite of the sub-cutaneous connective tissues which are actively transmitted through the bites of fleas/lice and ticks, respectively [15,16,17,18]. Furthermore, *A. dracunculoides* infests the peritoneal cavity of dogs [19] and a little known filarioid *Onchocerca lupi* inducing ocular nodules on the eyelids, conjunctiva and sclera in dogs as in human [20].

Some filarioids of the subfamilies Onchocercinae and Dirofilariinae are associated with an endosymbiotic intracellular bacterium of the genus *Wolbachia* [21], which can be found in all filarioid developmental stages and is essential for the long-term survival of the adult filarioids [22]. Moreover, *Wolbachia* are host-specific, and each filarial species that harbor *Wolbachia* is associated with a specific bacterial genotype [23]. These features constitute make its a suitable target for the diagnosis of filarial infection, especially when occurring in dead-end hosts as is the case of *D. immitis* in humans and cats [24,25]. Recent studies showed that the combined detection of *Wolbachia* and filarioid DNA improves the diagnosis of these infections [26,27].

CVBH are strongly related to the distribution of their vectors and are therefore geographically varied. Few studies data are available on canine filariasis from Latin America. The current species reported from canids in Brazil are potentially zoonotic and are often caused by *A. reconditum*, *C. bainae*, *D. grassii*, *D. immitis* and *D. repens* [28]. In addition, two cases of human filariasis have been reported across the continent, caused by lymphatic filariasis in Brazil, Dominican Republic, Guyana and Haiti and onchocerciasis in Brazil, Colombia, Ecuador, Guatemala, Mexico and Venezuela [29]. However, the information on CVBH in French Guiana is very scarce.

In the present study, we aimed to determine the presence of CVBH in dog blood samples from French Guiana as well as the strains of their endosymbiotic *Wolbachia* using molecular assays. Also aimed to investigating *D. immitis* infection rate using heartworm antigen test. The study provides preliminary information that could use in the future for the control of the potentially zoonotic filariasis in dogs from French Guiana.

## 2. Materials and Methods

### 2.1. Sampling and Study Area

In January 2016, in two sites of French Guiana separated by 60 km, 67 adult dogs, including 18 stray dogs and 49 shelter dogs were sampled in Cayenne (4°56′4.6″ N, 52°19′49.19″ W), the main city of French Guiana with 57,000 residents and 31 adult shelter dogs from Kourou (5°9′34.92″ N, 52°39′1.08″ W). All dogs were subjected to a blood sampling via a cephalic venipuncture using a BD Vacutainer™ K3EDTA tubes (Fisher Scientific, Illkirch, France), then conditioned at 4 °C and transported to our laboratory. Ethical aspects related to dog sampling were treated in accordance with the French law. Owner consent was obtained for all shelter dogs of both cities. Likewise, the consent of the director of the Cayenne dog pound was obtained for all stray dogs. All dogs (*n* = 98) were apparently healthy, including 52 females and 46 males.

### 2.2. Ethics Approval

Dogs were examined by veterinarians with the assistance and acceptance of their owners. Ethical aspects related to dog sampling were treated in accordance with the French law. The owner consents were obtained for all shelter dogs of both cities. Likewise, the consent of the director of the Cayenne dog pound was obtained for all stray dogs.

### 2.3. Microfilaria and Heartworm Antigen Tests

Immediately after sampling, the dog’s blood samples were processed for the detection of heartworm antigens using the WITNESS^®^ *Dirofilaria* (Zoetis, Lyon, France). According to the manufacturer’s recommendations, this immunochromatographic test allowed the identification of heartworm antigens from bloodstream. The blood wet mount preparation was also performed on each blood sample for microfilariae detection. One drop of homogenized EDTA blood was examined under a microscope at low magnification. Microfilaria-positive samples were confirmed by the visualization of live microfilariae moving like snakes between blood cells. In the present study, samples with *D. immitis* antigen-positive, but negative in PCR were considered as heartworm occult infection.

### 2.4. DNA Extraction

Genomic DNA was extracted individually from all blood samples. The detail of the extraction protocol is described elsewhere [9]. Briefly, DNAs were obtained after two lysis steps using a powder glass and proteinase K for the mechanical and enzymatic digestion, respectively. The extraction was carried out in Biorobot EZ1 System with the EZ1 DNA tissue kit (Qiagen, Courtaboeuf, France) following the manufacturer’s instructions. Each extraction was eluted in a total volume of 100 µL and stored at −20 °C until analysis.

### 2.5. Molecular Screening for Filarioid and Wolbachia DNAs

First, all samples were tested using the combined multiplex approach, recently developed for the detection of filarioid and *Wolbachia* DNAs [27]. The screening was processed as follows: (i) the exploration of filarial and *Wolbachia* DNAs using, respectively, the pan-filarial [Pan-Fil 28S] and the pan-*Wolbachia* [All-Wol-16S] qPCRs (Table 1), (ii) followed by species specific identification from samples tested positive for filarioid-*Wolbachia* DNAs using, respectively, the triplex [Triplex TaqMan COI] qPCR targeting *D. immitis*, *D. repens* and *A. reconditum* and the duplex [Wol-Diro *ftsZ*] qPCR targeting the prokaryotic homolog of the eukaryotic protein tubulin gene (*ftsZ*) of *Wolbachia* of *D. immitis* and that of *D. repens* (Table 1).

### 2.6. Molecular and Phylogenetic Characterization of Filarioid and Wolbachia

First, all samples that tested positive for filarial DNA were subjected to PCR amplification and sequencing analysis using the following systems: The standard (Pan-Nematoda) PCR [9] was used to amplify 1194 pb from the small subunit rRNA gene and the filarial specific (Pan-fil COI) PCR [27] targeting 509 pb from the Cytochrome c oxidase subunit I (*cox*1) gene. The third PCR was developed to amplify 497–570 pb from the 12S rDNA gene of filarial nematodes (Table 1), while all *Wolbachia*-positive samples were tested using the standard (W16S-Spec) PCR [30] targeting 438 pb fragment from the 16S rDNA.

When the filarial co-infections were found, we performed a serial 2-fold dilution of blood using Hank’s balanced salt solution (GIBCO^®^) followed by a DNA extraction as described above. This was performed in duplicate. The objective was to concentrate only one species of microfilariae before the extraction. Once, the DNA were obtained from each dilution they were processed for amplification using the 18S, *cox*1 and 12S PCR primers, then the last two positive dilution by each PCR were subjected to the sequencing analysis.

All PCR reactions were carried out in a total volume of 50 µL, consisting of 25 µL of AmpliTaq Gold master mix (Thermo Fisher Scientific, Waltham, MA, USA), 18 µL of ultra-purified water DNAse-RNAse free, 1 µL of each primer and 5 µL of genomic DNA. PCR reactions with all systems were run using the following protocol: incubation step at 95 °C for 15 min, 40 cycles of one minute at 95 °C, 30 s for the annealing at a different annealing temperature for each PCR assay and elongation from 45 s to 1 min and 30 s (Table 1) at 72 °C with a final extension for five minutes at 72 °C. PCR reactions were performed in a Peltier PTC-200 model thermal cycler (MJ Research, Inc., Watertown, MA, USA).

DNA amplicons generated through the PCRs were purified using filter-plate Millipore NucleoFast 96 PCR kit following the manufacturer’s recommendations (Macherey Nagel, Düren, Germany). Purified DNAs were subjected to the second reaction using the BigDye^®^ Terminator v. 3.1 Cycle Sequencing Kit (Applied Biosystems, Foster City, CA, USA), then the products were purified on the Sephadex G-50 Superfine gel filtration resin prior sequencing on the ABI Prism 3130XL.

Nucleotide sequences were assembled and edited by ChromasPro 2.0.0. The absence of co-amplification of nuclear mitochondrial genes (numts) was verified as recommended [31]. In addition, the visual verification of sequence chromatograms ambiguities, indels and stop codons of the translated sequences were performed by Chromas Pro 2.0.0 software. Sequences amplified from the filarial 18S, *cox*1 and 12S rDNA as well as the 16S rDNA of *Wolbachia* were subjected separately to a preliminary analysis using Basic Local Alignment Search Tool (BLAST) [32].

Filarial (18S, *cox*1 and 12S) and *Wolbachia* (16S) sequences obtained in this study were aligned with the closely related sequences retrieved from GenBank or Worm databases [33]. The alignment was performed using the ClustalW application within Bioedit v. 7.2.5. software [34]. DNA sequences of the non-filarial nematodes *Dracunculus medinensis (*AY852268), *Heliconema longissimum* (GQ332423) and (NC 016127) were used as outgroups for the 18S, *cox*1 and 12S trees, respectively and *Rickettsia* sp. (AB795333) for the *Wolbachia* 16S phylogram. Finally, the best nucleotide substitution model was chosen according to the Akaike Information Criterion (AIC) option in MEGA6 [35]. Phylograms were generated using the maximum likelihood (ML) method based on Kimura 2-parameter (+G) model [36] for both the 18S and the 16S and General Time Reversible (+G, +I) [37], Hasegawa-Kishino-Yano 5+G) models [38] for the *cox*1 and 12S phylograms, respectively. All phylograms were generated with 1000 bootstrap replicates using all sites of the sequences.

## 3. Results

**Figure 1:**
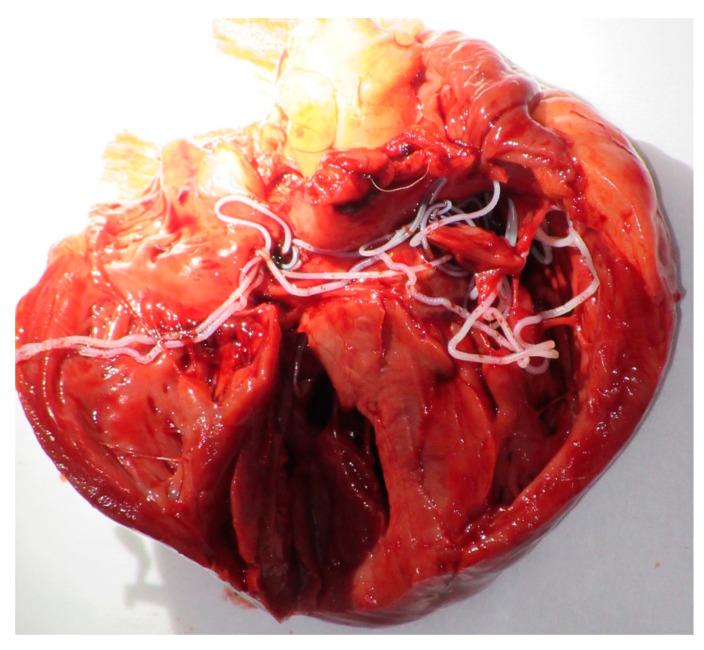
Heartworm (*Dirofilaria immitis*) in the canine heart of dog.

The detailed results of the dogs tested positive by at least one assay (parasitological, serologic or molecular) are shown in Table 2. The blood wet mount preparation revealed the presence of microfilariae in 6 (6.1%) samples. One of these dogs was a dangerous stray dog that had to be euthanized according to regulations. We performed the autopsy and reported 12 adult filariae (*D. immitis*) in the right ventricle (6 males and 6 females) (Figure 1). The heartworm antigen test detected 15 (15.3%) positive samples. The molecular screening revealed the presence of at least one molecular marker of filarioid and *Wolbachia* in 19 (19.4%) samples. Of these, 7 (7.1%) samples tested positive for both *Wolbachia* and filarial DNAs, 9 (9.2%) samples for filarial DNA only and 3 (3.1%) for *Wolbachia* DNA only.

Of those 16 dogs tested positive for filarial DNA, 11 (11.2%) and 3 (3%) samples were, respectively positive for *D. immitis* and *A. reconditum* by the triplex qPCR. Two samples remained unidentified by this assay. However, the duplex qPCR identified the specific DNA of *Wolbachia* endosymbiont of *D. immitis* in 6 (6.1%) samples. These latter were also positive for *D. immitis* DNA. However, the duplex qPCR did not amplify *Wolbachia* DNA from 4 samples. Compared to the molecular assays, the heartworm antigens were detected in 9 (9.1%) samples among those positive for *D. immitis* DNA and in 5 (6.1%) samples negative for *D. immitis* by qPCR. Of these latter, one sample was positive for *A. reconditum* DNA.

A nearly full-length DNA sequence of the 18S rDNA gene (1194 pb) was obtained from all samples tested positive for filarial DNA (*n* = 16). Filarioid single species DNA was obtained from 15 of them. The last one was found co-infected and yielded two amplicon sequences (Table S1).

Four genotypes were identified: 11 sequences of *D. immitis* (MN795071 to MN795081) were identical each other and showed 100% identity with *D. immitis* isolated from dogs in Japan (AB973231), 2 similar sequences of *C. bainae* (MN795085, MN795086) were identical to that isolated from dog in the USA (MH390715), 3 sequences of *A. reconditum* (MN795082, MN795083, MN795084) were 100% identical to that isolated from dogs in Côte d’Ivoire (MK495733). Of these, one sequence of *Brugia* sp. (MN795087) closely related to lymphatic filariasis with 99.9% identity with *Brugia malayi* (AF036588) and *Wuchereria bancrofti* (AY843436).

The *cox*1 and 12S sequences were obtained from all *D. immitis* and *A. reconditum* previously amplified by the 18S. *D. immitis cox*1 (MT193078 to MT193088) and 12S (MT252014 to MT252024) sequences were identical to each other for each gene and exhibited 99.78% and 100% of identity, respectively with *cox*1 (MT027229) and 12S (KF707482) sequences of *D. immitis* isolated from dogs, but were different from the virulent strain of *D. immitis* that occurred in Latin America [39]. This was observed for both *cox*1 and 12S, where identity was 91.71% and 95.12% with *cox*1 (HQ540424) and 12S (HQ540423), respectively. However, *A. reconditum cox*1 (MT193075 to MT193077) and 12S (MT252011 to MT252013) sequences showed an identity of 99.55% and 100% of identity with *A. reconditum cox*1 (JF461456) and 12S (AJ544853) sequences, respectively. The *cox*1 sequence of *Brugia* sp. was also amplified (MT193074) and displayed 93.39% identity with *B. timori* (AP017686), 92.73% with *B. malayi* (MK250713) and 91.19% with *B. pahangi* (MK250710). However, the *C. bainae* 12S sequences (MN795631, MN795631) showed, respectively 97.12% and 97% of identity and query cover with *C. bainae* (KF381408) isolated from dogs in Italy. Despite several attempts, the standard PCRs targeting the *cox*1 and the 12S rRNA gene failed to amplify the *C. bainae* and *Brugia* sp., respectively.

**Figure 2:**
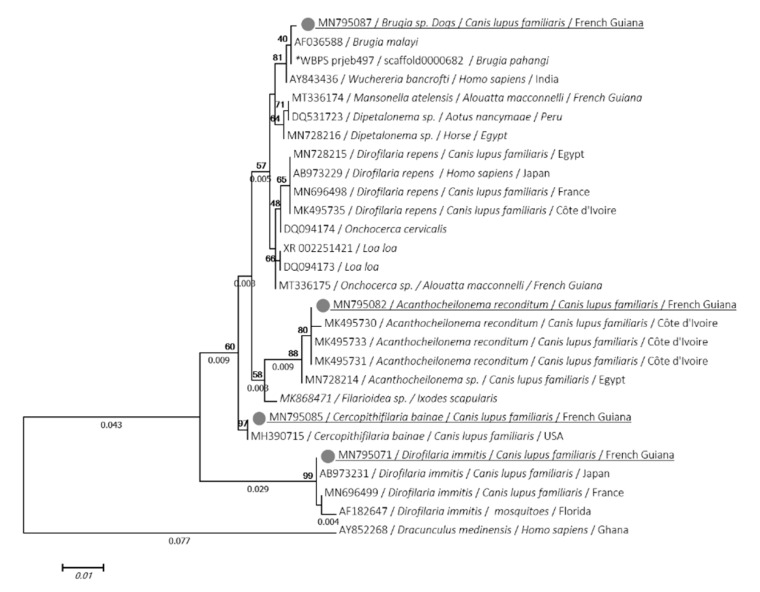
Phylogram of the 18S rRNA gene generated by maximum likelihood method from 28 partial (969) sequences. The tree with the highest log likelihood (−2078,7662) is shown. A discrete gamma distribution was used to model evolutionary rate differences among sites (5 categories (+G, parameter = 01254)). Numbers above and below branches are the display of bootstrap replicate values and branches length, respectively. Host, geographical location (when available) and GenBank accession number are indicated in each node. The sequences of the present study are underlined. *: indicates DNA sequences retrieved from Worm database.

**Figure 3:**
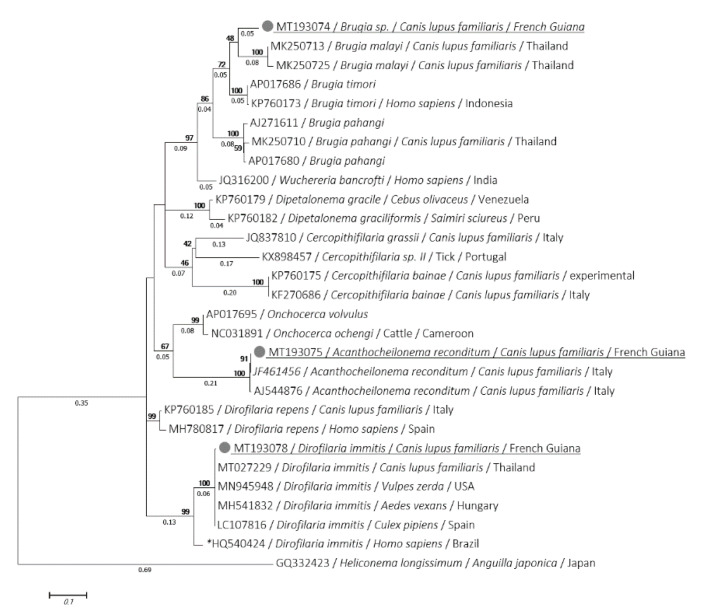
Phylogram of the *cox*1 gene generated by maximum likelihood method from 29 partial (449) sequences. The tree with the highest log likelihood (−2690.9892) is shown. A discrete Gamma distribution was used to model evolutionary rate differences among sites (5 categories (+G, parameter = 0.4403)). Numbers above and below branches are the display of bootstrap replicate values and branches length, respectively. Host, geographical location (when available) and GenBank accession number are indicated in each node. The sequences of the present study are underlined. *: indicates the most virulent strain of *D. immitis* from Latin America [39].

**Figure 4:**
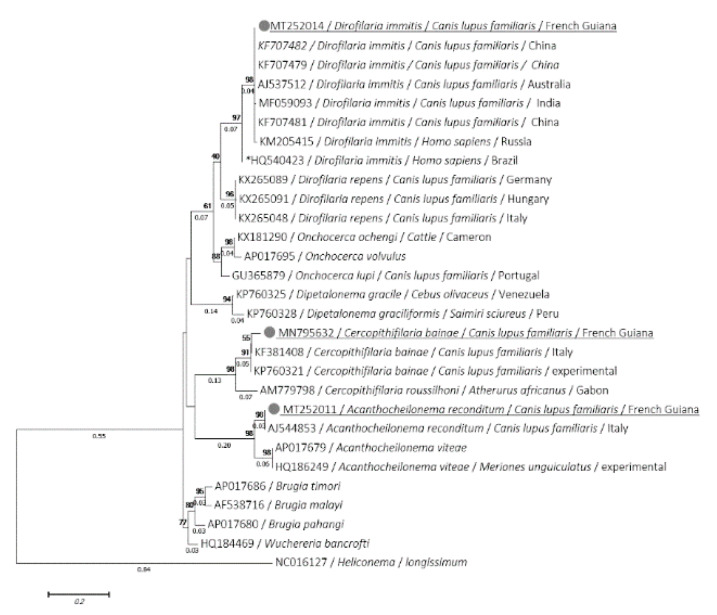
Phylogram of the 12S rRNA gene generated by maximum likelihood method from 29 partial (421) sequences. The tree with the highest log likelihood 5-2356.1682) is shown. A discrete Gamma distribution was used to model evolutionary rate differences among sites (5 categories (+G, parameter = 0.6437)). Numbers above and below branches are the display of bootstrap replicate values and branches length, respectively. Host, geographical location (when available) and GenBank accession number are indicated in each node. The sequences of the present study are underlined. *: indicates the most virulent strain of *D. immitis* from Latin America [39].

Phylogenetic analyses using ML method of the 18S, *cox*1 and 12S genes showed that the isolate of both *D. immitis* and *A. reconditum* from Guiana dogs clustered together with those usually isolated around the world (Figure 2, Figure 3 and Figure 4). Moreover, both *cox*1 and 12S trees (Figure 3 and Figure 4) indicate that *D. immitis* isolate is distinguished by a clearly separated branch from the virulent isolate from Brazil [39]. The isolate of *Brugia* sp. was clustered with brugian species and was a clearly separated branch although it was placed in the same genus. This was observed for both 18S (Figure 2) and *cox*1 inferences (Figure 3). While *C. bainae* isolates were clustered with the same species isolated from dogs in the USA (Figure 2). However, the 12S tree showed its separation from the isolated one in Europe (Figure 4).

**Figure 5:**
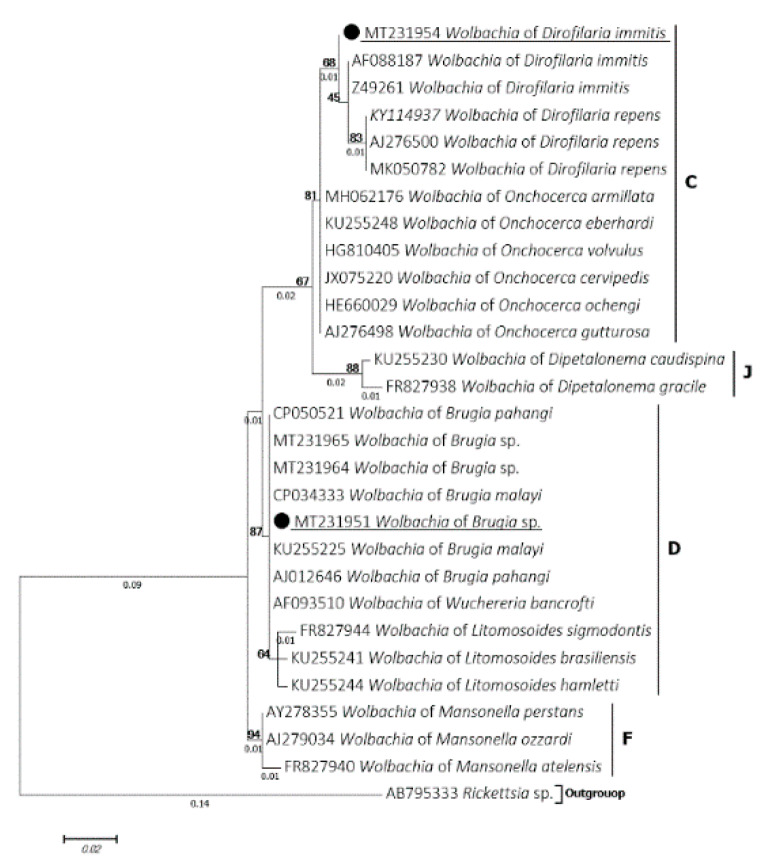
Phylogram of the *Wolbachia* 16S gene generated by maximum likelihood method from 29 partial (295) sequences. The tree with the highest log likelihood (−735.8600) is shown. A discrete Gamma distribution was used to model evolutionary rate differences among sites (5 categories (+G, parameter = 0.2679)). Numbers above and below branches are the display of bootstrap replicate values and branches length, respectively. Host, geographical location (when available) and GenBank accession number are indicated in each node. The sequences of the present study are underlined (black circle).

*Wolbachia* 16S partial sequences were successfully amplified and sequenced from all samples detected by qPCR. A total of 10 samples were amplified and were split into two distinct genotypes according to the BLAST results. Six identical sequences (MT231954, MT231955, MT231957, MT231958, MT231959, MT231960) showed an identity ranging from 99.3% to 99.6% with *Wolbachia* endosymbiont of *D. immitis* (MH062176, AF088187). These latter were isolated from samples positive for *D. immitis* single-specie-DNA. While three sequences were isolated from samples tested negative for filarial DNA and another one was obtained from a co-infected sample by *A. reconditum* and *Brugia* sp. All were identical to each other (MT231951, MT231952, MT231953, MT231956) and displayed 100% identity with the *Wolbachia* endosymbiont of *B. malayi* (CP034333), *B. pahangi* (AJ012646) and *B. timori* (AJ012646). The phylogenetic inference classified these genotypes into the clade C and D supergroups of *Wolbachia* and were clustered with *Wolbachia* endosymbiont of *D. immitis* and *Brugia* spp., respectively (Figure 5).

## 4. Discussion

This is the first molecular report of filaria and *Wolbachia* infections from dogs in French Guiana. Dogs are the most implicated reservoir for filariasis [9,10,11,15,17,19]. The control of these vector-borne helminths is based on epidemiological information and the use of adequate diagnostic methods [40]. The present study highlighted the presence of four filarial species in Guiana’s dogs: *D. immitis*, *A. reconditum* and for the first time *C. bainae* and *Brugia* sp. Compared to the parasitological assays, the molecular diagnosis remains the most adequate tool to study the diversity of these parasites [28]. This tool had gained an important increase in research area [41,42]. In addition, several studies have recently associated the molecular detection of *Wolbachia* in the diagnosis of canine filariasis [9,10,27].

Despite the several diagnostic assays we performed, our results remained not exhaustive and are limited by the sampling method. In the absence of skin samples, we are not able to achieve the exploration of filarial infections from Guiana’s dogs, especially those with cutaneous microfilariae, such as *Onchocerca lupi* and *Cercopithifilaria* spp. [17].

Microfilariae were not found in the blood samples of twelve dogs that tested positive for filarial DNA. The blood wet mount preparation is not a microfilariae concentration test, and only detects an integral microfilariae with a threshold of 30 microfilaria (mf)/mL [43,44]. Due to the lack of an adequate equipment in the field we were not able to perform a microfilariae concentration tests, such as Knott or filtration tests [45]. This represents a limitation of this study, which decrease the accuracy of our results in terms of sensitivity and specificity. Likewise, the molecular assays are able to detect the DNA from a micro-fragment of microfilaria with an analytical sensitivity threshold of 1.5 × 10^−4^ mf/mL [27].

It is worth highlighting that *D. immitis* infection is the most diagnosed canine filariasis in the world. In French Guiana, this parasite is known to be enzootic, as veterinarians often prescribe chemoprophylaxis. However, epidemiological data are lacking. The *D. immitis* infection rate of 16.3% (*n* = 98) reported here is complementary to a study carried out in Guyana using the Knott’s test (14.1%, *n* = 2135) [46] and elsewhere in Venezuela (15.2%, *n* = 138) and in Dominican Republic (18.2%, *n* = 104) [46,47].

In the field as in veterinary clinics, heartworm antigens tests are the most widely used assays. These tests detect the adult antigens of *D. immitis* from both occult and non-occult infections with different levels of sensitivity and specificity [48]. Here, the WITNESS^®^ Dirofilaria test revealed the presence of *D. immitis* antigens in 15 dogs, five of which were considered as an occult infection. Of these, one sample was also found positive for *A. reconditum* DNA. Despite the fact that this filarioid may cross-react on heartworm antigen tests [49], the occult infection cannot be excluded. In Latin America, *A. reconditum* and *D. immitis* are often present as co-infections in dogs [28]. Furthermore, the presence of *Spirocerca lupi* in Guiana dogs [50] could interfere with heartworm antigen and produce such results [41]. On the other hand, no antigens were detected from two *D. immitis* DNA-positive samples (CMT-34, CMT76) (Table 2). This could be related the presence of immune-complex that block the antigen detection process or to the low secretion of antigens by adults worms [8].

To the best of our knowledge, no human infection by *D. immitis* has been reported in French Guiana to date. Since humans can be infected, doctors could be confronted with this parasitosis, which is mainly characterized by the presence of pulmonary nodules. In South America, fifty sporadic cases have been reported: pulmonary (Venezuela, Colombia, Brazil, Argentina) and subcutaneous/ocular nodules (Brazil, Chile) [8,51,52]. *D. immitis* implicated in human ocular dirofilariasis was classified as a more virulent strain [51]. Fortunately, the phylogenetic analysis we conducted indicated that it is not the virulent *D. immitis* (Figure 3 and Figure 4) which could explain the lower prevalence of human cases [39].

It is not surprising that no dog was found parasitized by *D. repens* responsible for subcutaneous dirofilariasis [34]. This benign, but more zoonotic dirofilariasis is only described in the Old World. In America, a few doubtful cases of *D. repens* were reported [53,54,55] wherein a lack of information was provided on the origin of the dogs examined. The possibility that this is an imported case cannot be ruled out.

*A. reconditum* is the causative agent of canine subcutaneous filariasis [15]. This affection, characterized by subcutaneous nodules, is largely neglected as it usually remains unnoticed with no clinical consequences The infection has a worldwide distribution, including the United States, South America, Oceania and many African and European countries [15]. Human infections by *A. reconditum* are quite exceptional. One case (subconjunctival infection) has been described in Australia [36] and two cases in Turkey [56].

Surprisingly, we found the DNA of *C. bainae* in the blood of two dogs. This filarioid was first described in Brazil and has since been reported in Europe, Africa, Australia and Americas [57]. The adult form of these tick-borne filaroids usually dwell beneath the cutaneous tissues of infected dogs, while their microfilaria are distributed unevenly in superficial dermal tissues [58]. Our study constitutes the first report of its DNA in blood. The study conducted by Rojas et. al. (2015), reported the presence of a filarioid DNA from the blood, wherein a low-quality inclusive DNA sequence of a dermal filarioid was detected using the HRM real time qPCR. The same dog was skin-positive for *C. bainae* DNA [57]. These results are not exhaustive and further investigation of this filarioid from skin samples are needed to describe the real prevalence of this filariasis. In addition, our results are complementary to the phylogenetic analysis of *C. bainae* carried out on isolates from Brazil, which revealed the evolutionary separation of isolates from Europe and Latin America [59].

Another surprising finding was the detection of a filarioid species from the genus *Brugia*. Canine brugian infections are often caused by *Brugia* species associated with lymphatic filariasis in Asia (*B. malayi, B. pahangi* and *B. timori*) [12]. While the other brugian infections are encountered in wild animals, such as the Asian primates, raccoons and rabbits in the USA [60]. Recently, in Brazil, blood microfilaria from the genus *Brugia* were reported from the ring-tailed coatis (*Nasua nasua*) [61]. In Guiana, the unique species of *Brugia* was described morphologically from the lymphatic system of the coatimundi (*Nasua nasua vittata*) and was named *Brugia guyanensis* (Orihel 1964) [62]. The possibility that the *Brugia* sp. herein we detected is the same *B. guyanensis* cannot be ruled out in the absence of morphologic identification. Our findings highlighted the circulation of a potential zoonotic *Brugia* in French Guiana dogs. In addition, human health could be at risk if the nematode reported here in domestic dogs had zoonotic potential.

Finally, our results reported the presence of two *Wolbachia* strains from the clade C and D of filarial nematodes, wherein the first one was clustered with those associated with *D. immitis* and the second one with those of lymphatic filarial parasites. In terms of host-relationship, *Wolbachia* strain is filaria species-specific [23], which allows them to serve as a diagnostic target [10,24,45]. Moreover, in heartworm infected dogs and cats, these bacteria are implemented in pulmonary disease [24,25]. Clarifying these bacteria by antibiotic treatments induces infertility and death of filarioid worms and has reduced the incidence of inflammatory pulmonary lesions and thrombi associated with heartworm disease, providing an effective treatment strategy for the control and eradication of the filarial infection in human and dogs [22,26].

## 5. Conclusions

The present study molecularly detected *D. immitis*, *A. reconditum*, *C. bainae* and *Brugia* sp. and the associated *Wolbachia* endosymbionts from canine blood in French Guiana. *Brugia* sp. and *C. bainae* were detected for the first time in French Guiana dogs. It would be interesting to know whether *Brugia* sp. DNA we detected corresponds to a new species or if it is *Brugia guyanensis*. To this end, a morphologic based-taxonomy should be investigated simultaneously with molecular studies in the future. In addition, further studies based on blood and skin samples are required to expand the epidemiological knowledge of these nematodes in French Guiana. Finally, there is an urgent need for the implementation of preventive chemoprophylaxis against these vector-borne helminths. The use of the anti-*Wolbachia* drugs should also be explored in the future.

## Figures and Tables

**Table 1:** Primers and probes used in this study.

Application	System Name’s	Target Gene	Primer & Probes Name’s	Sequences 5′–3′	Amplicon Size (pb)	Tm/Elongation Time	Specificity	References
qPCR	Pan-Fil 28S	28S LSU rRNA	qFil-28S-F	TTGTTTGAGATTGCAGCCCA	151	60 °C/30″	Filarial species	[27]
			qFil-28S-R	GTTTCCATCTCAGCGGTTTC				
			qFil-28S-P	6FAM-5′-CAAGTACCGTGAGGGAAAGT-3′-TAMRA				
	All-Wol-16S	16S rRNA gene	all.Wol.16S.301-F	TGGAACTGAGATACGGTCCAG	177	60 °C/30″	*Wolbachia* sp.	
			all.Wol.16S.478-R	GCACGGAGTTAGCCAGGACT				
			all.Wol.16S.347-P	6FAM-5′-AATATTGGACAATGGGCGAA-3′-TAMRA				
	Triplex TaqMan COI	*cox*1	Fil.COI.749-F	CATCCTGAGGTTTATGTTATTATTTT	166	60 °C/30″	FAM: *D. immitis* VIC: *D. repens* Cy5: *A. reconditum*	
			Fil.COI.914-R	CWGTATACATATGATGRCCYCA				
			D.imm.COI.777-P	6FAM-CGGTGTTTGGGATTGTTAGTG-TAMRA				
			D.rep.COI.871-P	6VIC-TGCTGTTTTAGGTACTTCTGTTTGAG-TAMRA				
			A.rec.COI.866-P	Cy5-TGAATTGCTGTACTGGGAACT-BHQ-3				
	Wol-Diro ftsZ	Prokaryotic homolog of the eukaryotic protein tubulin	WDiro.ftsZ.490-F	AAGCCATTTRGCTTYGAAGGTG	111	60 °C/30″	FAM: *Wolbachia* of *D. immitis* VIC: *Wolbachia* of *D. repens*	
			WDiro.ftsZ.600-R	AAACAAGTTTTGRTTTGGAATAACAAT				
			WDimm.ftsZ.523-P	6FAM-CGTATTGCAGAGCTCGGATTA-TAMRA				
			WDrep.ftsZ.525-P	6VIC-CATTGCAGAACTGGGACTGG-TAMRA				
PCR	16S W-Spec	16S rRNA	W-Specf	CATACC TATTCGAAGGGATAG	438	60 °C/1′	*Wolbachia* sp.	[30]
			W-Specr	AGCTTCGAGTGAA ACCAATTC				
	Pan-Nem 18S	18S SSU rRNA	Fwd.18S.631	TCGTCATTGCTGCGGTTAAA	1127–1155	54 °C/1′30″	Nematodes	[9]
			Rwd.18S.1825r	GGTTCAAGCCACTGCGATTAA				
	Pan-Fil *cox*1	*cox*1	Fwd.957	ATRGTTTATCAGTCTTTTTTTATTGG	509	52 °C/45″	Filarial species	[27]
			Rwd.1465	GCAATYCAAATAGAAGCAAAAGT				
	Pan-Fil 12S	12S rRNA	Fwd.12S.110	TCCAGAATAATCGGCTATACATTTT	497 to 570	56 °C/45″	Filarial species	The present study
			Rwd.12S.681	CCATTGACGGATGGTTTGTA				

**Table 2:** Results of dogs positive for at least one assays (parasitologic, serologic and molecular assays).

Sample Description		Parasitology	Serology	Filarioid							*Wolbachia*				Decision
				qPCR-Based Detection			Multi-Locus Genotyping				qPCR-Based Detection		16S Genotyping		
Dog Code	Location	Thin Smear	Witness *Dirofilaria*	Filarial DNA	*D. imm*	*A. rec*	Accession Number (AN)			Species	*Wolbachia*	WDim	16S (AN)	*Wolbachia* Clade/Strains	
							18S	*Cox*1	12S						
CMT 01	Cayenne	Neg.	Neg.	Pos.	N/A	Pos.	MN795082 MN795087	MT193075 MT193074	MT252011	*A. reconditum Brugia* sp.	Pos.	N/A	MT231951	*D/WBr.*	*A. reconditum Brugia* sp.
CMT 12	Cayenne	Neg.	Neg.	N/A	N/A	N/A	..-..	..-..	..-..	*..-..*	Pos.	N/A	MT231952	*D/WBr.*	*Brugia* sp.
CMT 13	Cayenne	Neg.	Neg.	N/A	N/A	N/A	..-..	..-..	..-..	*..-..*	Pos.	N/A	MT231953	*D/WBr.*	*Brugia* sp.
CMT 14	Cayenne	Neg.	Pos.	Pos.	N/A	Pos.	MN795083	MT193076	MT252012	*A. reconditum*	N/A	N/A	..-..	..-..	*A. reconditum/* O-Heartworm
CMT 18	Cayenne	Pos.	Neg.	Pos.	N/A	Pos.	MN795084	MT193077	MT252013	*A. reconditum*	N/A	N/A	..-..	..-..	*A. reconditum*
CMT 19	Cayenne	Pos.	Pos.	Pos.	Pos.	N/A	MN795071	MT193078	MT252014	*D. immitis*	Pos.	Pos.	MT231954	*C/WDim.*	*D. immitis*
CMT 32	Kourou	Neg.	Pos.	N/A	N/A	N/A	..-..	..-..	..-..	..-..	N/A	N/A	..-..	..-..	O-Heartworm
CMT 34	Kourou	Neg.	Neg.	Pos.	Pos.	N/A	MN795072	MT193079	MT252015	*D. immitis*	N/A	N/A	..-..	..-..	*D. immitis*
CMT 36	Kourou	Neg.	Pos.	N/A	N/A	N/A	..-..	..-..	..-..	*..-..*	N/A	N/A	..-..	..-..	O-Heartworm
CMT 38	Kourou	Neg.	Pos.	Pos.	Pos.	N/A	MN795073	MT193080	MT252016	*D. immitis*	N/A	N/A	..-..	..-..	*D. immitis*
CMT 40	Kourou	Pos.	Pos.	Pos.	Pos.	N/A	MN795074	MT193081	MT252017	*D. immitis*	N/A	N/A	..-..	..-..	*D. immitis*
CMT 41	Kourou	Neg.	Pos.	Pos.	Pos.	N/A	MN795075	MT193082	MT252018	*D. immitis*	Pos.	Pos.	MT231955	*C/WDim.*	*D. immitis*
CMT 43	Kourou	Neg.	Neg.	N/A	N/A	N/A	..-..	..-..	..-..	*..-..*	Pos.	N/A	MT231956	*D/WBr.*	*Brugia* sp.
CMT 52	Kourou	Neg.	Pos.	Pos.	Pos.	N/A	MN795076	MT193083	MT252019	*D. immitis*	N/A	N/A	..-..	..-..	*D. immitis*
CMT 53	Kourou	Neg.	Pos.	N/A	N/A	N/A	..-..	..-..	..-..	*..-..*	N/A	N/A	..-..	..-..	O-Heartworm
CMT 54	Kourou	Neg.	Pos.	N/A	N/A	N/A	..-..	..-..	..-..	*..-..*	N/A	N/A	..-..	..-..	O-Heartworm
CMT 61	Cayenne	Neg.	Pos.	N/A	N/A	N/A	..-..	..-..	..-..	*..-..*	N/A	N/A	..-..	..-..	O-Heartworm
CMT 71	Cayenne	Pos.	Pos.	Pos.	Pos.	N/A	MN795077	MT193084	MT252020	*D. immitis*	Pos.	Pos.	MT231957	*C/WDim.*	*D. immitis*
CMT 75	Cayenne	Pos.	Pos.	Pos.	Pos.	N/A	MN795078	MT193085	MT252021	*D. immitis*	Pos.	Pos.	MT231958	*C/WDim.*	*D. immitis*
CMT 76	Cayenne	Neg.	Neg.	Pos.	Pos.	N/A	MN795079	MT193086	MT252022	*D. immitis*	N/A	N/A	..-..	..-..	*D. immitis*
CMT 89	Cayenne	Neg.	Neg.	Pos.	N/A	N/A	MN795085	..-..	MN795631	*C. bainae*	N/A	N/A	..-..	..-..	*C. bainae*
CMT 90	Cayenne	Neg.	Neg.	Pos.	N/A	N/A	MN795086	..-..	MN795632	*C. bainae*	N/A	N/A	..-..	..-..	*C. bainae*
CMT 91	Cayenne	Pos.	Pos.	Pos.	Pos.	N/A	MN795080	MT193087	MT252023	*D. immitis*	Pos.	Pos.	MT231959	*C/WDim.*	*D. immitis*
CMT 97	Cayenne	Neg.	Pos.	Pos.	Pos.	N/A	MN795081	MT193088	MT252024	*D. immitis*	Pos.	Pos.	MT231960	*C/WDim.*	*D. immitis*

Neg.: negative, Pos.: positive, N/A: no amplification, AN: Accession number, D. imm: *D. immitis*, A. rec: *A. reconditum*, WBr: *Wolbachia* endosymbiont of *Brugia* sp., WDim: *Wolbachia* endosymbiont of *D. immitis*, O-heartworm: occult heartworm.

## Supplementary Materials

The following are available online at https://www.mdpi.com/2076-2607/8/5/770/s1, Table S1: PCR/Sequencing results of the 18S.
