# Supplementary material for: Detection of Canine Vector-Borne Filariasis and Their Wolbachia Endosymbionts in French Guiana
Source: Microorganisms. 2020 May 21;8(5):770. doi: 10.3390/microorganisms8050770 (PMC7285362; doi:10.3390/microorganisms8050770)
Supplement: Supplementary file 1 [file microorganisms-08-00770-s001.pdf]

| Dilution | 18S | | Cox1 | | 12S | |
|---|---|---|---|---|---|---|
| | R-1 | R-2 | R-1 | R-2 | R-1 | R-2 |
| 1/400 | Pos[a] | Pos. | Pos[a] | Pos | Pos[a] | Pos[a] |
| 1/800 | Pos[a] | Pos[a] | Pos[a] | Pos[a] | Pos[a] | Pos[a] |
| 1/1600 | N/A | Pos[b] | N/A | Pos[b] | N/A | N/A |

Pos: Sample positive by PCR. a: *A. reconditum*. b: *Brugia* sp. N/A: No amplification, R-1 and R-2 represents duplicate 1 and 2 respectively.

**Table S1.** PCR/Sequencing results of the 18S, cox1 and 12S genes from a filarial co-infected sample (CMT1) after microfilaria separation using a serial 2-fold dilution of blood.
